# Latent Membrane Protein 1 of Epstein–Barr Virus Promotes RIG-I Degradation Mediated by Proteasome Pathway

**DOI:** 10.3389/fimmu.2018.01446

**Published:** 2018-06-28

**Authors:** Chongfeng Xu, Lei Sun, Wenjun Liu, Ziyuan Duan

**Affiliations:** ^1^Genetic Resources Center, Institute of Genetics and Developmental Biology, Chinese Academy of Sciences, Beijing, China; ^2^CAS Key Laboratory of Pathogenic Microbiology and Immunology, Institute of Microbiology, Chinese Academy of Sciences, Beijing, China

**Keywords:** RIG-I, LMP1, nasopharygeal carcinoma, Epstein–Barr virus cancer, immunotherapy of cancer

## Abstract

RIG-I signaling is critical to host innate immune response against RNA virus infection, and also can be activated against many kinds of cancer. Oncogene LMP1 of Epstein–Barr virus (EBV) contributes to various tumors progress. In this study, we have provided strong evidence that LMP1 inhibits Sendai virus mediated type I interferon production and downregulates RIG-I signaling pathway by promotion RIG-I degradation dependent on proteasome. Nineteen kinds of E3 ligase are identified by IP-MS as LMP1-interactors, they are candidate E3s, which are possibly recruited by LMP1 to mediate RIG-I degradation. CHIP is among these E3s, which has been reported to lead RIG-I degradation. Notably, we find C666-1, an EBV-positive nasopharyngeal carcinoma cell line, expresses low level of RIG-I, even treated with IFN-α, RIG-I expression could not be induced. This evidence indicates that EBV employs a unique strategy to evade RIG-I mediated immune responses.

## Introduction

Epstein–Barr virus (EBV), a gamma-herpesvirus, establishes lifelong asymptomatic infections in the majority of population worldwide (1). Although largely harmless, the virus is also causally associated with lymphoproliferative disease, infectious mononucleosis, and is related to a broad spectrum of malignancies of lymphoid and epithelial origin such as nasopharyngeal carcinoma (NPC), gastric cancer, and even breast cancer (2–4). EBV infection is accompanied by extensive cellular changes caused by the interaction of the invasion and host defense.

Innate immunity is an essential part of host defense system and pattern recognition receptor is the first line of innate immunity. Retinoic acid-inducible gene I (RIG-I) and melanoma differentiation associated protein 5 (MDA5) are two essential cytosolic receptors for detection of virus-derived RNAs in the cytoplasm. Besides its critical roles of antiviral immunity, more and more studies suggest that activation of RIG-I can facilitate therapies of many kinds of cancer (5–16).

Upon virus infection or treatment with agonist, the helicase domains of RIG-I/MDA5 detect virus-derived RNAs, their caspase activation and recruitment domain (CARD) are released and RIG-I/MDA5 becomes activated, the active RIG-I/MDA5 then interacts with mitochondrial antiviral signaling protein (MAVS), a down-stream mitochondrial adaptor molecule *via* RIG-I CARD and MAVS CARD interactions. Consequently, MAVS becomes active to stimulate signaling effectors TBK1 (TANK-binding kinase 1) or IKK (inhibitor-κB kinase), which activate transcription factor IFN-regulatory factors IRF-3 and/or IRF-7 and NF-κB, respectively. Activated IRFs and NF-κB are translocated into the nucleus, and interact with the promoter regions of target genes, including IFNs and inflammatory cytokines (17). Type I interferon (IFN-α/β) system has a very important role in controlling viral infection by promoting the synthesis of multiple antiviral proteins like ISG15 (18).

Both antiviral and antitumor activity of RIG-I depend on functional RIG-I induction. Functional RIG-I can not only be induced at protein expression level but also can be activated from inactive state by RNA virus, agonist, or IFN-α treatment (9). RIG-I has been reported to be regulated by EBV infection. EBER, the non-coding RNA encoded by EBV can activate RIG-I to induce IFNs on EBV-positive Burkitt’s lymphoma cell line (19, 20), but little is known about whether RIG-I functions well in NPC. In this study, we found that LMP1 downregulated the Sendai virus (SeV) and RIG-I stimulated IFN-β production, and further identified that LMP1 promotes RIG-I degradation, interestingly, the expression level of RIG-I on NPC cell line C666-1, which possesses EBV genome, is significantly lower than EBV negative NPC cells and could not be induced by IFN-α. This evidence indicates that EBV has evolved a unique strategy to evade RIG-I mediated immune responses, and this reminds us to considerate the therapies based functional RIG-I may be hampered by LMP1.

## Materials and Methods

### Cell Lines and Antibodies

NP69 (immortalized human nasopharyngeal epithelial cell line, EBV negative), CNE2 (NPC cell line, EBV negative), Hone1 (NPC cell line, EBV negative), C666-1 (only available EBV positive NPC cell line) were kindly provided by Zuguo Li (Southern Medical University, Guangzhou) (21), NP69 was cultured with Defined K-SFM medium, CNE2, Hone1 and C666-1 were maintained in 1640 medium, 293T and human amnion WISH cells was cultured in DMEM medium (life technology), all kinds of cell except NP69 were supplemented with 10% FBS (Gibco-life technology), and incubated at 37°C in 5% CO_2_. Antibodies and their manufacturers were: rabbit mAb anti-RIG-I (D14G6, 3743S) was from Cell-Signaling Technologies, rabbit anti-ISG15 (EPR3446) and rabbit monoclonal to IRF3 phospho S386 (ab76493) were from Abcam. Mouse monoclonal anti-GAPDH (60004-1-Ig, Proteintech), mouse anti-FLAG clone M2 (F1804), mouse anti-c-Myc (C3956), anti-FLAG agarose affinity gel (A-2220), and 3xFLAG peptide (F4799) were Sigma products; HRP (horseradish peroxidase-conjugated) conjugated secondary antibodies were from Jackson.

### SeV Infection

Sendai virus was gifted from Liu W (22). For virus infection of cells, the culture medium was removed from the plates, and the cells were washed twice with PBS. Serum free culture medium containing SeV (MOI = 1) was added for 2 h, the medium was removed, and washed with PBS twice carefully, disturbing cells were avoided, and then medium containing 2% FBS culture medium was added.

### Plasmids

The promoter luciferase reporter plasmids IFN-β-Luc, NF-kB-Luc, IFN-stimulated response element (ISRE)-Luc, and expression plasmids RIG-I, RIG-IN, MDA5C, MAVS, TBK1, IRF3/5D were provided by Liu W (22) (Chinese Academy of Sciences, China). Plasmids TRAFD1 (RC200265) and CHIP (RC200310) were products of OriGene company.

#### IFN Assay

The ability of IFN to reduce the cytopathic effects (CPE) of vesicular stomatitis virus (VSV, gifted from Prof. Liu W) on WISH cells was assayed. 293T cells were transfected with pCMV-Myc-LMP1, 24 h post transfection, infected with SeV, after 1 h absorption of SeV, wash 293T cells carefully. After 6 or 12 h infection, supernatants of cell were collected. Serial fourfold dilutions of supernatants from LMP1-expressing cells (vector as control) were added in 0.1 ml volumes to WISH monolayers in 96 microtiter wells. After 12 h incubation, the medium was removed and VSV in 0.1 ml (1000 TCID50) was added to each well. When there was a complete CPE in the virus control cultures, usually within 30 h, the cultures were rinsed with PBS, fixed, and stained with crystal violet formaldehyde-ethanol solution. One unit of the IFN titer was determined as the highest dilution that inhibited at least 50% of the CPE. Interferon titer was calculated with Reed–Muench method.

### Immunoprecipitation and Immunoblotting

For immunoblotting and co-immunoprecipitation, cells harvested 48 h post transfection were lysed in lysis buffer containing 0.5% NP40, 150 mM NaCl, 20 mM HEPES (pH 7.4), 10% glycerol, 1 mM EDTA, and protease inhibitor cocktail. Protein concentration was measured with a protein assay kit (Bio-Rad Laboratories). Co-immunoprecipitation was performed as describe previously (23), the cell lysates were incubated overnight with anti-FLAG agarose affinity gel followed by washing with lysis buffer and elution with 3×-FLAG peptide, respectively, at a concentration of 500 µg/ml. Immunocomplexes were washed with lysis buffer and elution was performed by boiling for 10 min in 2× SDS-PAGE loading buffer. 8, 10, or 12% SDS-PAGE gels were used to separated different proteins, after transferring proteins to polyvinylidene difluoride (PVDF) membranes (Millipore), the blots were blocked in Tris-buffered saline containing 5% non-fat milk and 0.1% Tween 20 and incubated with primary antibodies either for 1 h at room temperature or overnight at 4°C followed by incubation for 1 h with the appropriate HRP secondary antibodies. The complexes were visualized by chemiluminescence.

### Luciferase Assay

293T cells were seeded into 24-well plates. The following day, cells were transfected with 200 ng luciferase plasmid and 100 ng β-Gal plasmid, along with 200–400 ng plasmids required for different experiments. 24 h later, cells were lysed in lysis buffer. After centrifugation, the supernatants were stored at −80°C. The luciferase assays were performed with a luciferase assay kit (Promega).

### Co-Immunoprecipitation

Co-IP was also used for examination of specific interactions between the LMP1 and E3 ligases. Meanwhile, 293T cells were transfected with plasmids expressing Myc-tagged LMP1 or control vectors with FLAG-Myc-TRAFD1, or CHIP for 24 h, and the cell lysates were collected and incubated with an anti-FLAG M2 affinity gel at 4°C for 6 h. After mixing and incubation, the immunoprecipitated proteins were eluted with SDS-PAGE sample buffer. For immunoblotting, the cell extracts and the cellular proteins co-purifying with the FLAG-Myc-TRAFD1 or FLAG-Myc-CHIP protein were separated by SDS-PAGE and then transferred to the PVDF membranes. The membranes were then probed with anti-Myc, anti-FLAG, antibodies.

### CHX, MG132, and NH4CI Treatment

Cells were treated with 100 mg/ml CHX for various periods of time after transfection. MG132 (10 mM) and NH4Cl (10 mM) were used at the same time as CHX, and cells were harvested 3 h after treatment.

## Results

### LMP1 Negatively Regulates SeV and RIG-I-Mediated Activation of Interferon Signaling Pathway

**Figure 1:**
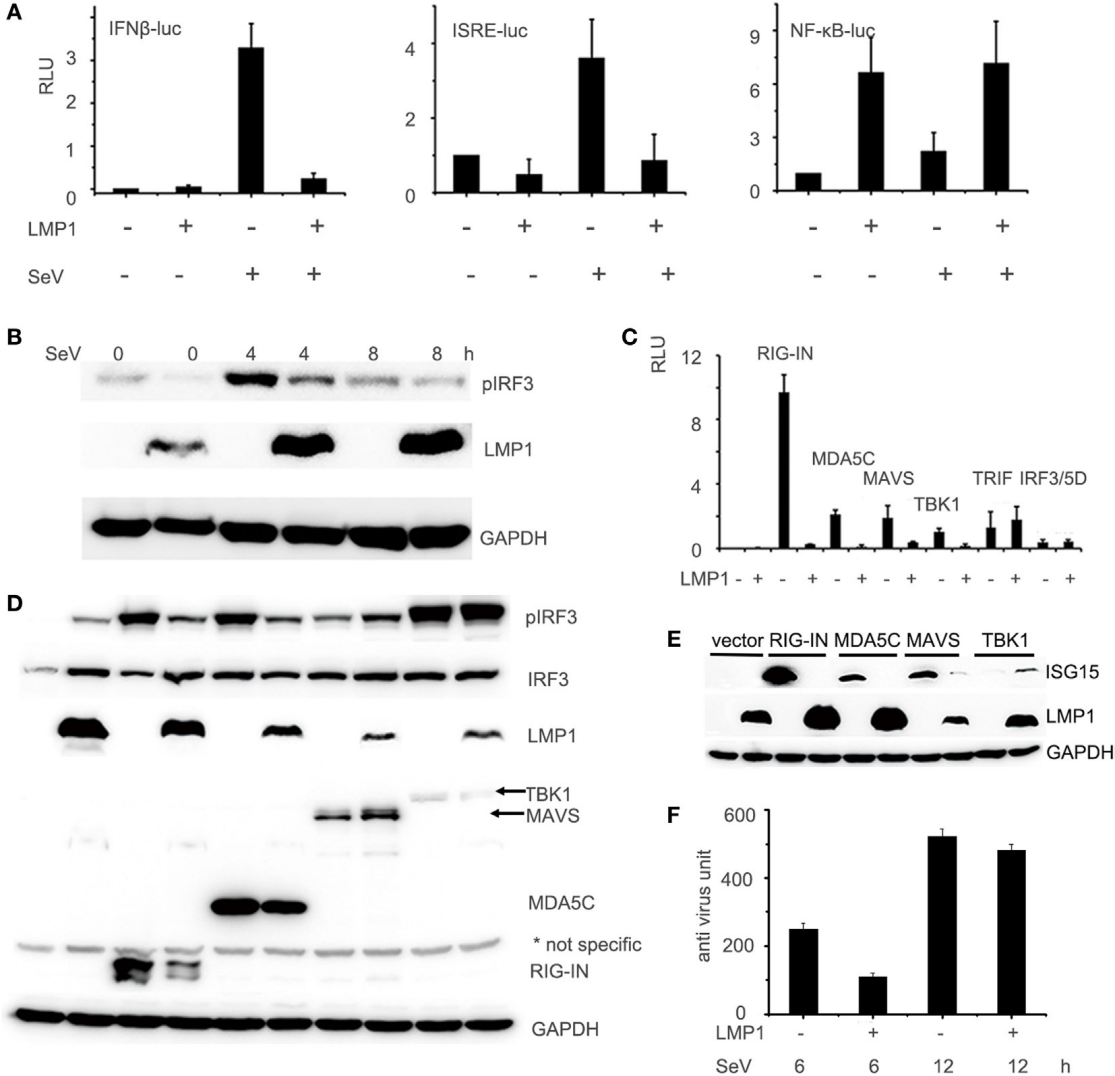
LMP1-inhibits SeV and RIG-I-mediated transactivation of IFN-β. **(A)** 293T cells were transfected with 0.1 µg IFN-β, ISRE, or NF-κB luciferase reporter together with 0.1 µg pCMV-β-gal (as internal control), and 0.5 µg pCMV-Myc-LMP1 or empty vector. 24 h after transfection, 293T cells were challenged with SeV for 12 h. Luciferase activity was analyzed at 12 h post-infection and relative luciferase activity (RLU) (relative to the basal level of reporter gene in the presensence of pCMV-Myc vector after nomalization with co-transfected β-gal activity) was determined compared to uninfection control. The data are shown as the mean ± SD and are representative of one of three independent experiments (*n* = 3). Results are representative of at least three experiments run in triplicates. **(B)** CNE2 cells were transfected with pCMV-LMP1, after 24 h transfection, infected by SeV for 4 and 8 h, phosphorylated IRF3 of CNE2 cells was detected by Western blot. **(C)** 293T cells were transfected with 0.5 µg pCMV-LMP1 (vector as control), 0.25 µg signal molecules 0.1 µg IFN-β and 0.1 µg β-gal. Luciferase activity was analyzed after 24 h post transfection. **(D)** 293T cells were transfected with 0.25 µg signal molecules, pEGFP-IRF3, and pCMV-LMP1 (vector as control). 24 h post transfection, phosphorylated IRF3 was detected by Western blot. **(E)** 293T cells were transfected with 0.25 µg signaling molecules and pCMV-LMP1 (vector as control). 24 h post transfection, endogenously induction of ISG15 was detected by Western blot. **(F)** 293T cells were transfected with 1 µg pCMV-LMP1 (vector as control) per well in 6-well plates, stimulated with SeV (MOI = 1) for 6 and 12 h, supernatants were collected to test IFNs antivirus titers. IFNs activity was determined by IFN assays: calculating the protection of the WISH cells against the cytopathic effects caused by VSV.

With the IFN-β, ISRE, and NF-κB promoter reporter systems, we evaluated the effect of LMP1 on SeV stimulated IFN signaling pathway. As Figure 1A showed, over-expression of LMP1 significantly impaired SeV-mediated activation of IFN-β and ISRE promoters, but not NF-κB, which is well known to be activated by LMP1. To further investigate whether LMP1 has inhibitory activity on type I interferon production in other type of cells, we evaluated it on CNE2 cells, an EBV negative NPC cell line. As demonstrated in Figure 1B, over-expression of LMP1 also inhibited SeV-induced IRF3 phosphorylation. RIG-I is an important receptor response to SeV infection, in order to explore the inhibition mechanisms of LMP1 imputed on the SeV-mediated activation of IFN-β signal pathway, we over-expressed the active form of RIG-I signaling moleculars, which is N terminal of RIG-I, C terminal of MDA5, MAVS, TBK1, and IRF3/5D (constitutively active IRF-3) to stimulate IFN-β reporter (Figure 1C), the phosphorylation of IRF3 (Figure 1D), and the expression of IFN-β stimulate gene 15 (ISG15) (Figure 1E), we found that LMP1 inhibited RIG-I/MAVS-mediated transactivation of IFN-β promoter, IRF3 phosphorylation, and ISG15 production. To check the actual effect of inhibition of LMP1 on SeV-mediated IFNs production, we have tested the concentration of IFNs in the supernatants of SeV stimulated cells expressing LMP1 or not. Antiviral activity of interferons in the supernatants was determined by IFN activity of protection WISH CPE against VSV. As Figure 1F showed, at 6 h after stimulation of SeV, IFN production of the LMP1-expressing cells is less than half of its level in the LMP1 absent control, late stimulation at 12 h, the gap of IFNs production between LMP1-expressing and control become small. All the data collectively suggested that LMP1 negatively regulated RIG-I signaling pathway. Besides these results, compared with no LMP1-expression control, it appeared that the expression of RIG-I and MDA5 were reduced by over expression of LMP1 (Figure 1D).

### LMP1 Promotes RIG-I Degradation Dependent on Proteasome Pathway

**Figure 2:**
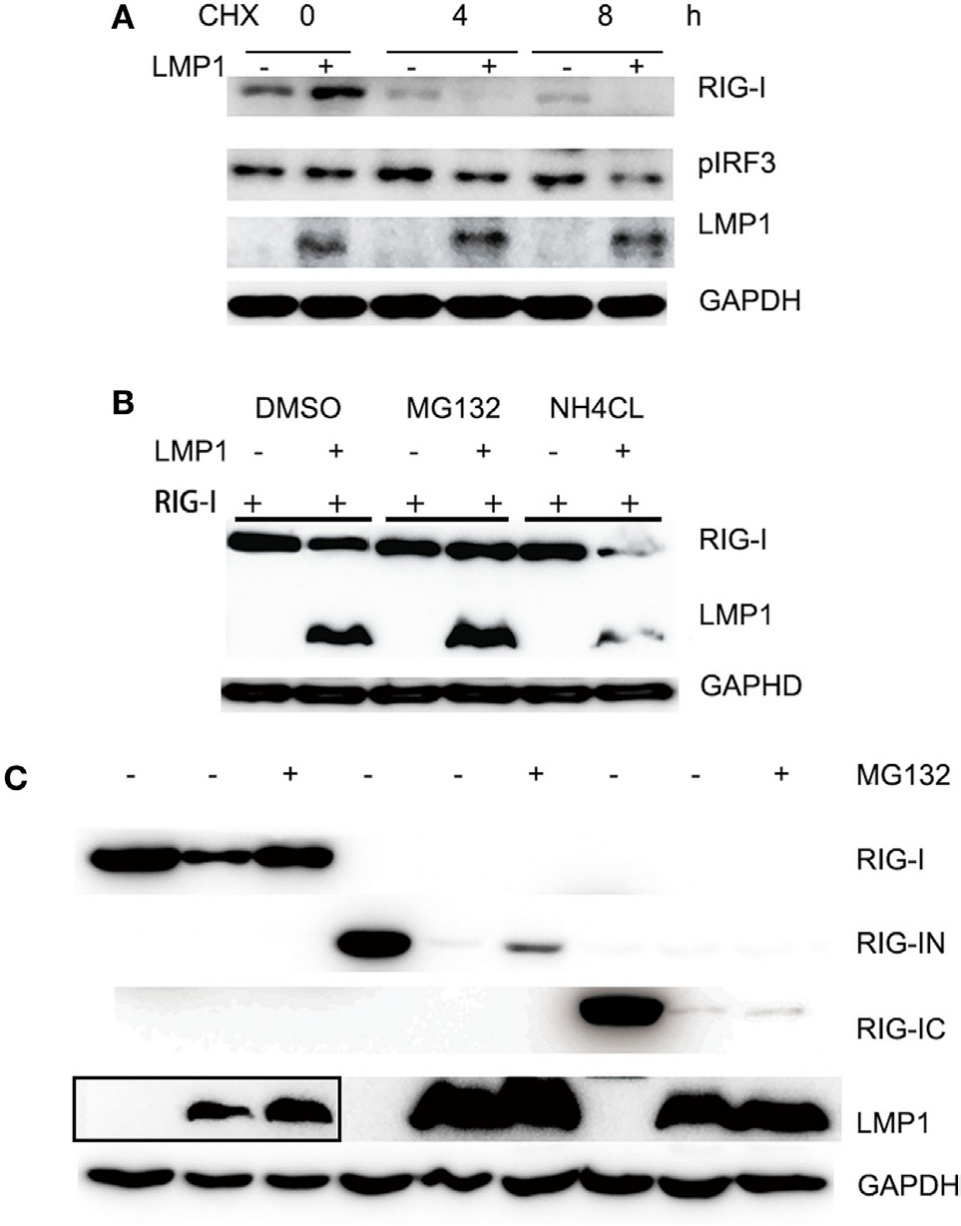
LMP1-promotes degradation of RIG-I and MG132 inhibited RIG-I degradation. **(A)** Immunoblot analysis of endogenously induced RIG-I and phosphorylated IRF3 in CNE2 cells. CNE2 were transfected with pCMV-LMP1 (empty vector as control) for 24 h, then infected with Sendai virus for 4 h, after infection, CNE2 was treated with CHX (100 mg/ml) for 3 h, RIG-I and pIRF3 of cell lysates were analyzed with Western blot. **(B)** Immunoblot analysis of FLAG tagged RIG-I in 293T cell lysates, which were transfected with pCMV-LMP1 (empty vector as control) and pcDNA-FLAG-RIG-I for 16 h, then treated with MG132 or NH4CL for 3 h. **(C)** FLAG tag was probed for immunoblot analysis of RIG-I and its N terminal and C terminal truncates in 293T cell lysates, which were co-transfected with FLAG-RIG-I/RIG-IN/RIG-IC and pCMV-LMP1 (vector as control) for 16 h, then treated with MG132 for 3 h. The Myc-LMP1 line was from two images, which were pictured from the same polyvinylidene difluoride film with different exposure time, the box area exposure time was longer than the right part.

During test the effect of LMP1 on RIG-I signaling pathway, we found that the RIG-I protein level in LMP1-expressing group is significantly lower than vector control (Figure 1D). In consideration of LMP1 and RIG-I expressing vector with CMV promoter we used, it is possible that the low protein level of RIG-I is the result of LMP1 that influences the stability of mRNA or protein of RIG-I. With quantitative real-time PCR, we found no significant different mRNA level between vector and over-expression of LMP1. To further determine whether LMP1 influenced the protein stability of RIG-I, we transfected LMP1 in CNE2, then infected CNE2 with SeV to induce RIG-I expression, 4 h after SeV infection, added CHX to inhibit new protein synthesis, and we found that LMP1 could promote degradation of endogenous expressed RIG-I (Figure 2A). To determine which protein degradation pathway was responsible for RIG-I degradation promoted by LMP1, proteasome inhibitor MG132 and lysosome inhibitor NH4CL were used to test their protection activity from RIG-I degradation. Results showed that MG132 could inhibit LMP1-mediated RIG-I degradation, while, lysosome inhibitor NH4CL had no inhibition activity on RIG-I degradation promoted by LMP1 (Figure 2B). These data indicated that LMP1 promoted RIG-I degradation depending on proteasome pathway. The domain of RIG-I, which can be degraded was also evaluated, over-expression of LMP1 and domains of RIG-I, 16 h post transfection, MG132 was added to treat 293T cells for 3 h, the expression of RIG-I and its partial truncations were analyzed with Western blot. Results demonstrated that LMP1 could promote full length RIG-I, N terminal and C terminal of RIG-I degradation, and MG132 has partially inhibitory activity on their degradation (Figure 2C).

### LMP1 Interacts With E3 Ligase CHIP

**Figure 3:**
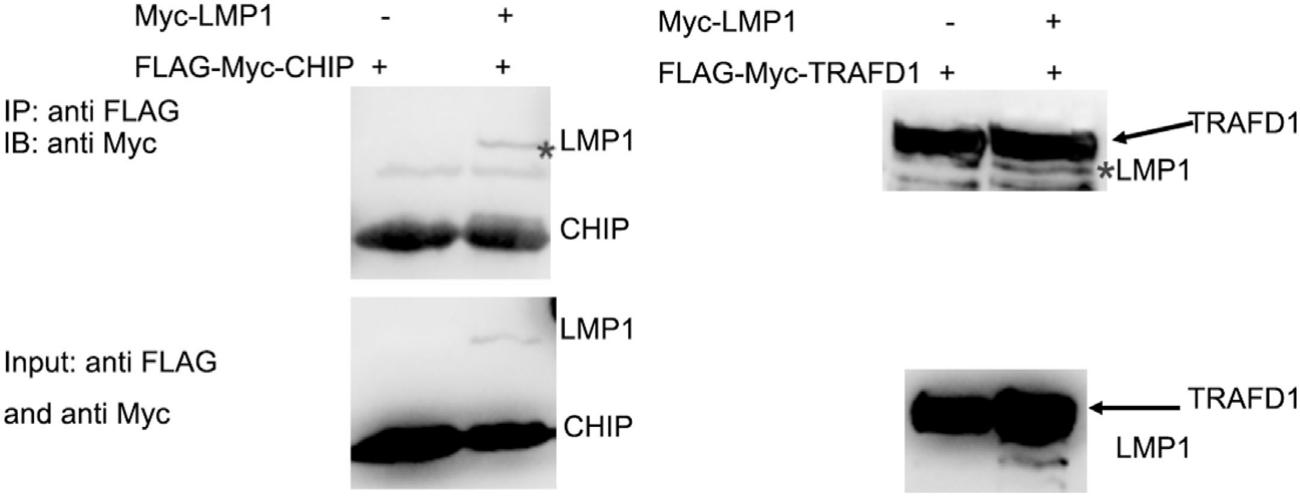
LMP1 interacts with E3 ligases CHIP and TRAFD1. The 293T cells were transfected with plasmids E3 ligases FLAG-Myc-CHIP (MW: 28 kD) or FLAG-Myc-TRAFD1(MW: 65 kD) with Myc-LMP1 (MW: 55kD). After transfection of 24 h, Co-IP was performed with cell lysates with antibodies to FLAG tags. Immunoblot analysis of Myc-LMP1-immunoprecipitated by FLAG-tagged E3 with antibodies to c-Myc. Upper panel shows the IP results performed with anti-FLAG monoclonal antibody, and immunoblot analysis with anti Myc. The lower panel shows the input, immunoblot with anti-FLAG and anti-Myc monoclonal antibodies. * represents immunoprecipitated Myc-LMP1.

It is E3 ligase that interacts with the target protein and makes its substrate polyubiquitination with Lys48-linked chains of ubiquitin, targeting the substrate for destruction by the proteasome. Given that LMP1 has no E3 enzyme activity, it is reasonable that LMP1 recruits E3 ligases to RIG-I and promoted RIG-I degradation. To find E3 ligases interacted with LMP1, we have explored co-immunoprecipitation and mass spectrometry (IP-MS) by over-expressing FLAG tagged LMP1. Among the proteins co-immunoprecipited with LMP1, 19 kinds of E3 ligases including CHIP (Table 1) were identified as LMP1-interacting proteins in 293T cells (Figure 3; Table 1). These ubiquitin ligases may participate in ubiquitination of cytosolic pattern recognition receptor RIG-I. Two E3 ligases (CHIP and TRAFD1) were used to validate the interaction between LMP1 and E3s identified by the IP-MS (Figure 3). In the test, FLAG-Myc-CHIP (MW: 28 kD) or FLAG-Myc-TRAFD1 (MW: 65 kD) and Myc-LMP1 (MW: 55kD) were co-transfected into 293T cells, and the Co-IP was performed with an anti-FLAG affinity gel. The results show that both CHIP and TRAFD1 have undergone Co-IP with LMP1 (Figure 3), suggesting that LMP1 may utilize these E3 ligases to modify cell functions by ubiquitination.

**Table 1:** The E3 ligases interact with LMP1-identified by IP-MS.

Accession number	Gene	*P* value	Peptide	Number of peptide (*P* < 0.05)/identified peptides
Q9UNE7	CHIP	0.031	ALELDGQSVK	2/7
		1.10E−05	LNFGDDIPSALR	

Q14669	TRIP12	2.90E−05	LLDTNPEINQSDSQDSR	1/13

P49792	RANBP2	5.10E−05	ELLQSFDSALQSVK	1/20

O94822	LTN1	0.011	AASSQLR	2/7
		0.0014	LVNLADCLCNEDLESR	

Q9C0C9	UBE2O	0.00087	GLQEGYENSR	1/7

O75592	MYCBP2	0.0027	DLALPIGNELEEDLEILEEAALQVCK	1/24

Q9BV68	RNF126	0.015	CESGFIEELPEETR	1/4

Q9HCM9	TRIM39	0.018	ALLGLVK	2/5
		0.019	ALLGLVK	

Q9ULT8	HECTD1	2.80E−05	QDCSQLVER	6/21
		9.10E−06	ETSSLESFVR	
		0.0011	CPFLIPFETR	
		0.0015	EAASQRPLSSSASNR	
		3.00E−06	SELPDSIESALQGDER	
		0.00029	SCGLFTAPFPQDSDELER	

Q7Z6Z7	HUWE1	0.0001	ADGTATAPPPR	8/36
		0.0025	TPTEAPADCR	
		0.00061	DSAVAISGADSR	
		2.30E−06	VSDGGSSSTDFK	
		0.0012	AIQDPAFSDGIR	
		9.90E−05	SAATSGAGSTTSGVVSGSLGSR	
		0.016	SSDPLGDTASNLGSAVDELMR	
		0.018	SSDPLGDTASNLGSAVDELMR	

Q9UKV5	AMFR	0.058	SWLEQDTSCPTCR	4/7
		1.50E−07	SWLEQDTSCPTCR	
		0.0059	SSEDDAASESFLPSEGASSDPVTLR	
		7.40E−08	ALSQPEAGPGEPDQLTASLQPEPPAPARPSAGGPR	

Q12933	TRAF2	0.0012	GPNDALLR	9/16
		0.0047	HLEHECPER	
		1.30E−08	LDQDKIEALSSK	
		1.50E−05	LDQDKIEALSSK	
		0.0038	LDQDKIEALSSK	
		0.00037	LDQDKIEALSSK	
		0.0002	TATFENIVCVLNR	
		4.60E−06	GTLKEYESCHEGR	
		1.10E−05	EVESLPAVCPSDGCTWK	

Q13114	TRAF3	4.10E−06	VTELESVDKSAGQVAR	3/13
		8.00E−09	VTELESVDKSAGQVAR	
		8.10E−05	SAGTPVFVPEQGGYKEK	

O14545	TRAFD1	0.00026	TELCGNCGR	4/11
		1.20E−07	LSNSDSQDIQGR	
		0.00071	AVCEADQSHGGPR	
		0.016	LDSQPQETSPELPR	

Q9BY78	RNF26	0.029	LALGSEAWR	1/3

Q7L0R7	RNF44	0.0053	LGDAKPR	1/3

Q96PM5	RCHY1	0.0093	ICESYNTAQAGGR	1/1

Q13263	TRIM28	1.80E−06	VLVNDAQKVTEGQQER	1/8

O60337	MARCH6	0.016	AAEELTWER	1/2

### Low Expression of RIG-I on EBV Positive NPC Cell Line C666-1

**Figure 4:**
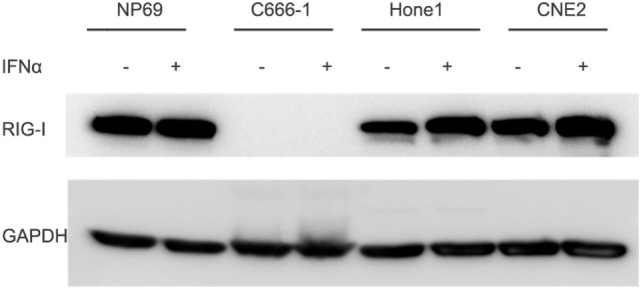
Expression of RIG-I is low in C666-1 cells and could not be induced by IFN-α. Nasopharyngeal epithelial cell line NP69 and nasopharyngeal carcinoma cell lines were cultured in the absence or presence of 100 U/ml of IFN-α for 12 h. Expression of RIG-I was determined by Western blot analysis.

Functional RIG-I can be activated against some kinds of tumors, to evaluate whether there is functional RIG-I in NPC cell lines, different human nasopharyngeal epithelial cell lines were cultured in the absence or presence of 100 IU/ml IFN-α for 12 h, expression of RIG-I was determined by Western blot analysis. Result demonstrated three patterns of RIG-I expression response to IFN-α treatment in three different kinds of nasopharyngeal epithelial cells (Figure 4). NPC cell lines Hone1 and CNE2 expressed basal levels of RIG-I that were upregulated upon IFN-α treatment. Nasopharyngeal epithelial cell line NP69 also expressed basal RIG-I; however, no increase of expression upon IFN-α treatment. Of note, NPC cell line C666-1, which consistently harbors EBV expressed significantly lower level of RIG-I than other nasopharyngeal epithelial cells, and IFN-α could not upregulate RIG-I expression. These results suggest that there is no functional RIG-I in EBV positive NPC.

## Discussion

Viral gene products initiate cellular changes by interfering with a variety of cellular signaling pathways. Our findings give us a new perspective on the capacity of the LMP1 encoded by human herpesvirus EBV to downregulate an innate antiviral response by promoting the RIG-I degradation. Previously studies have demonstrated LMP1-mediated activation of NF-κB is sufficient for LMP1-mediated priming to produce robust levels of endogenous IFNs upon infection of SeV in DG75 cells (24, 25). However, by exploring the reporters of IFN-β, ISRE on 293T cells, and verified the results on NPC cell lines, our study provided strong evidence that LMP1 inhibited SeV-mediating activation of IFN-β by promoting RIG-I degradation, therefore, inhibited host antiviral innate immunity. The deep mechanisms of the different phenomenon are still needed to be explored.

Here, we uncover a key role for LMP1 in the regulation of RIG-I stability depending on proteasome pathway. Many viruses have evolved strategies to evade host innate immune responses by causing RIG-I degradation (26, 27), several E3 ligases are involved in RIG-I degradation (28–30). LMP1 does not possess the E3 ligase activity, to identify the candidate E3s, which are seized by LMP1 to promote RIG-I degradation, we have performed co-immunoprecipitation and mass spectrometry to identify E3 ligases interacting with the LMP1, 19 kinds of E3 were identified, of which, we find CHIP, which was reported to mediated RIG-I degradation, so that CHIP likely mediates RIG-I degradation enrolled by LMP1, the other E3s are also possibly responsible for degradation of RIG-I. These considerations are mostly speculative, and the detail mechanisms by which LMP1 promoted RIG-I degradation are required to elucidate.

It is remarkable that the expression of RIG-I is lower in EBV-positive NPC cell line C666-1 than other NPC cell lines. It is important to note that C666-1 is the only EBV-positive NPC cell lines, so whether EBV infection lead to low expression of RIG-I need to be tested further. The expression of RIG-I may also be interfered by other molecules encoded by EBV, such as miR-BART6-3p negatively regulated the RIG-I targeting the 5′ UTR of RIG-I mRNA (31). On the other side, EBV infection-induced RIG-I expression is reported by several studies in EBV-positive Burkitt’s lymphoma or classical Hodgkin lymphoma cells (20, 32), while in NPC cells and specimens from NPC patients, the transcription level of RIG-I was associated with EBER transcription in a dose-dependent manner. These studies have described the mRNA level of RIG-I, but not compared the protein level of RIG-I in EBV-positive NPC relative to EBV-negative cell lines. EBV infection complicates the expression of RIG-I, so whether LMP1 is the principle reason of the extremely low RIG-I protein level of C666-1 is needed to be examined.

In a word, our findings on the interplay between LMP1 and RIG-I have important implications for EBV-associated NPC therapy. First, we provide a new cellular target of the oncogene and suggestion therapies targeting LMP1 have multiple effects. Second, our data indicate that expression level and the inducible capability of functional RIG-I vary considerably in different NPC cells. Recent publications have highlighted therapeutic implications of RIG-I agonists as antitumor immunotherapy agents (33). Given RIG-I antiviral activity and ligands-mediated antitumor responses depend on functional RIG-I expression, this treatment should be a careful assessment especially for EBV-positive NPC, because RIG-I might contribute to NPC development (34) or RIG-I is destroyed by EBV and could not be induced by IFN-α as revealed in this study. Finally, it is likely that additional immunosuppressive mechanisms employed by LMP1 contribute to excluding innate immunity from cancers. Clarifying these mechanisms may help develop new strategies for EBV-associated cancer immunotherapy.

## Author Contributions

CX and ZD conceived the experiments, CX conducted the experiments, CX, LS, and WL analyzed the results. CX wrote the paper and all authors reviewed the manuscript.

## Conflict of Interest Statement

The authors declare that the research was conducted in the absence of any commercial or financial relationships that could be construed as a potential conflict of interest.
